# Simultaneous two-color X-ray absorption spectroscopy using Laue crystals at an inverse-compton scattering X-ray facility

**DOI:** 10.1107/S1600577521009437

**Published:** 2021-11-03

**Authors:** Juanjuan Huang, Benedikt Günther, Klaus Achterhold, Martin Dierolf, Franz Pfeiffer

**Affiliations:** a Chair of Biomedical Physics, Department of Physics, School of Natural Sciences, Technical University of Munich, 85748 Garching, Germany; b Munich Institute of Biomedical Engineering, Technical University of Munich, 85748 Garching, Germany; c Department of Diagnostic and Interventional Radiology, School of Medicine, Klinikum Rechts der Isar, Technical University of Munich, 81675 München, Germany; d Institute for Advanced Study, Technical University of Munich, 85748 Garching, Germany

**Keywords:** energy-dispersive X-ray absorption spectroscopy, Laue optics, bimetallic systems

## Abstract

X-ray absorption spectroscopy (XAS) is an element-selective technique that provides electronic and structural information of materials and reveals the essential mechanisms of the reactions involved. However, the technique is typically conducted at synchrotrons and usually only probes one element at a time. In this paper, a simultaneous two-color XAS setup at a laboratory-scale synchrotron facility is proposed based on inverse Compton scattering (ICS) at the Munich Compact Light Source (MuCLS), which is based on inverse Compton scattering (ICS). The setup utilizes two silicon crystals in a Laue geometry. A proof-of-principle experiment is presented where both silver (Ag) and palladium (Pd) *K*-edge X-ray absorption near-edge structure spectra were simultaneously measured. The simplicity of the setup facilitates its migration to other ICS facilities or maybe to other X-ray sources (*e.g.* a bending-magnet beamline). Such a setup has the potential to study reaction mechanisms and synergistic effects of chemical systems containing multiple elements of interest, such as a bimetallic catalyst system.

## Abstract 2

A proof-of-principle simultaneous two-color XAS experiment on a sample containing both silver and palladium at a laboratory-scale synchrotron facility based on inverse Compton scattering is presented.

## 1. Introduction

Bimetallic catalysts, which consist of two metal components rather than a single host metal, form a major class of heterogeneous catalysts (Somorjai & Li, 2010 ▸; Tao, 2012 ▸). Adding the second metal can often enhance catalytic performance in terms of selectivity, activity and stability due to the synergistic effects between the two metals (Sankar *et al.*, 2012 ▸; Toshima & Yonezawa, 1998 ▸). For example, silver–palladium (Ag–Pd) bimetallic catalysts have been used for catalytic processes such as hydrogenation (Choi *et al.*, 2019 ▸; Tedsree *et al.*, 2011 ▸; Zhang *et al.*, 2000 ▸), de­hydrogenation (Guo *et al.*, 2003 ▸; Huang *et al.*, 2010 ▸), oxidation (Tou *et al.*, 2019 ▸) and reduction reactions (Li *et al.*, 2016 ▸). Probing multiple specific metallic element sites is essential for a better understanding of the interplay between the two metals in the bimetallic chemical systems.

Since the development of synchrotron X-ray user facilities, X-ray spectroscopy techniques, such as X-ray absorption spectroscopy (XAS) and X-ray emission spectroscopy (XES), have become powerful tools for various research fields (Bergmann & Glatzel, 2009 ▸; Lytle, 1999 ▸). Both XAS and XES are element-specific spectroscopic methods, and they provide valuable information about the geometric and electronic structures of materials. Though the development of modern X-ray spectroscopy methods enables high-speed data acquisition, *e.g.* down to milliseconds for quick-scanning X-ray absorption fine structure (Uruga, 2017 ▸), the X-ray spectroscopy techniques generally only probe one element at a time. In other words, recording full XAS/XES spectra of multiple elements is a sequential process. Ideally, data from different metals in the bimetallic system should be detected in parallel to eliminate errors induced from sample variability, radiation damage, *etc*. Especially for an *in situ* or *in operando* experiment at a short time scale, simultaneous detection of multiple metal centers makes it possible to track synchronous chemical changes and synergistic effects in the bimetallic systems. There are only a few setups discussed below that are capable of parallel data acquisition.

Simultaneous detection of multiple elements has been recently realized by XES employing curved crystals in von Hamos geometry (Gul *et al.*, 2015 ▸; Kalinko *et al.*, 2020 ▸) and crystal analyzers in Rowland geometry (Finkelstein *et al.*, 2016 ▸; Martinie *et al.*, 2018 ▸). In these setups, all elements in the sample are illuminated by the same incident X-ray beam. As outgoing X-rays with different energies are emitted to a 4π sterad solid angle, multiple spectral analyzers can be placed at different positions to detect emission lines from multiple elements in parallel.

In XAS, only a few publications report simultaneous measurement of two elements, realized using a dispersive geometry. This geometry utilizes a polychromator, which introduces a correlation between the propagation direction and energy of the X-ray beam (Pascarelli *et al.*, 2006 ▸). Different energies can be resolved in a single shot by a position-sensitive detector. To probe multiple elements at a time, the geometry and the bending radius of the polychromator can be adjusted so that the dispersive energy range is large enough to cover multiple absorption edges. Such setups have been applied to bimetallic catalytic systems such as Pt–Ge (Fiddy *et al.*, 2001 ▸) and Au–Pt particles (Nayak *et al.*, 2017 ▸). As the setup only uses one dispersive optics, the energy range is restricted to a relatively small energy window. For example, the 0.2 m-long Si(111) polychromator used by Fiddy *et al.* (2001 ▸) could cover ∼1000 eV for X-ray energy at ∼10 keV. Also, the measurement condition cannot be optimized independently for each element. Another dispersive XAS system utilizing two polychromators was recently reported (Katayama *et al.*, 2020 ▸). The setup is more sophisticated than the previous ones: the incident X-ray is first split by a double-hole mask and then dispersed separately by two polychromators. Two incident X-rays are focused at the same sample position for spectra acquisition. This setup was used to study a Ni–Cu bimetallic catalyst and a LiNi_0.5_Mn_1.5_O_4_ electrode.

Most XAS/XES experiments, including the two-color XAS/XES experiments discussed above, were demonstrated at large synchrotron facilities, owing to their superior performance (*e.g.* high brilliance and energy tunability of X-rays) compared with conventional X-ray tubes. However, the limited access to the large facilities and constantly oversubscribed beam time (for example, the oversubscription for all beamlines at the European Synchrotron Radiation Facility was 275% in 2018 according to its annual highlight report of 2019) prevent XAS/XES experiments from being conducted on a routine basis. For this reason, the last decade has witnessed intensive and significant development of modern laboratory XAS/XES setups using X-ray tubes (Seidler *et al.*, 2014 ▸; Németh *et al.*, 2016 ▸; Bès *et al.*, 2018 ▸; Błachucki *et al.*, 2019 ▸; Ditter *et al.*, 2019 ▸; Schlesiger *et al.*, 2020 ▸; Zeeshan *et al.*, 2020 ▸). Recent review articles (Zimmermann *et al.*, 2020 ▸; Malzer *et al.*, 2021 ▸) have provided detailed overviews of the laboratory setups and their applications. Among these laboratory setups, one interesting implementation is reported by Błachucki *et al.* (2019 ▸), which can simultaneously acquire XAS and XES at the same time using two von Hamos crystals as dispersive optics. They demonstrated simultaneous acquisition of *K*-edge XAS and *K*β XES of iron, nickel and copper metal foils. However, this setup focuses on simultaneous XAS and XES detection on the same element, not the two-element simultaneous scheme we want to present in the manuscript.

The laboratory setups are more efficient in the X-ray regime of about 5 to 12 keV than in the higher-energy regimes, owing to the significantly reduced flux of bremsstrahlung and reduced efficiency of employed optics at the high energies. For X-ray energies above ∼12 keV, inverse Compton scattering (ICS) sources have emerged as one of the most promising types of compact X-ray sources. For example, the Munich Compact Light Source (MuCLS), located at the Technical University of Munich, can provide an integrated X-ray flux of more than 10^10^ photons s^−1^ in a bandwidth (BW) of less than 5%, and correspondingly a spectral brilliance of 1.2 × 10^10^ photons s^−1^ mm^−2^ mrad^−2^ (0.1% BW)^−1^ in the range of 15–35 keV (Günther *et al.*, 2020 ▸). Previously, we have demonstrated a first proof-of-principle XAS experiment on the Ag *K*-edge (∼25.5 keV) conducted at the MuCLS facility (Huang *et al.*, 2020 ▸) with an acquisition time down to 1 min. The ICS facilities offer a complimentary regime to the 5–12 keV laboratory setups.

In this work, we present a two-color XAS setup based on two Laue crystals at the MuCLS facility. Laue crystals have been widely used in many applications, especially for high-energy X-rays. Apart from X-ray spectroscopy (Lecante *et al.*, 1994 ▸; Jagodziński *et al.*, 2019 ▸; Pascarelli *et al.*, 2016 ▸; Qi *et al.*, 2019 ▸; Szlachetko *et al.*, 2013 ▸), Laue crystals have been used for, for example, *K*-edge subtraction imaging (Zhu *et al.*, 2014 ▸), multiple energy X-ray imaging (Bassey *et al.*, 2016 ▸) and X-ray beam expanding (Martinson *et al.*, 2014 ▸).

The two-color XAS setup proposed here is straightforward to implement, since, apart from the X-ray source, it is mainly composed of two standard silicon (Si) wafers and one charge-coupled device (CCD) camera. The two crystals can be independently adjusted according to the respective energy range and energy resolution requirements. We performed a proof-of-principle two-color XAS experiment and simultaneously measured Ag as well as Pd *K*-edge X-ray absorption near-edge structure (XANES) with good spectral qualities. The implementation at the MuCLS has the potential for studying synergistic effects between Ag and Pd in the Ag–Pd bimetallic systems, or other element pairs in the desired energy range. In combination with our ongoing efforts in *in situ* XAS developments at our ICS facility on campus (Huang *et al.*, 2021 ▸), which is especially advantageous when sample transfer to synchrotron facilities is challenging or multiple iterations of an experiment are necessary, the proposed setup may unravel new opportunities for studying interaction mechanisms — time-resolved or *in situ* — between multimetallic sites involved in catalytic processes.

## 2. Experimental

**Figure 1:**
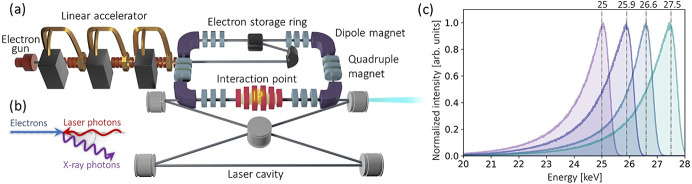
(*a*) Schematic of the inverse Compton scattering (ICS) source of the Munich Compact Light Source (MuCLS) facility. (*b*) Inverse Compton scattering between high-energy electrons and infrared photons generates X-rays at the interaction point. (*c*) Energy tunability of the source: exemplary MuCLS spectra at 25, 26, 27 and 28 keV energy configurations. The peak energies of X-ray source spectra deviate from the exact integer number due to slightly different X-ray tuning conditions/parameters than the defined ones.

The MuCLS is a versatile laboratory X-ray facility (Günther *et al.*, 2020 ▸). The X-rays at the MuCLS are generated by an ICS source (Lyncean Technologies Inc., Fremont, USA), shown in Fig. 1 ▸(*a*). In the ICS process [Fig. 1 ▸(*b*)], electrons and laser photons collide head-on at the interaction point, giving rise to the generation of high-energy X-rays. X-ray beam divergence is constrained by an output aperture to 4 mrad × 4.5 mrad (horizontal × vertical). Currently, the X-ray energy can be tuned between 15 and 35 keV by varying the electron energy. Fig. 1 ▸(*c*) shows exemplary MuCLS spectra at four different energy configurations (measured with a KETEK AXAS-D detector, KETEK GmbH, Munich, Germany). The high spectral flux density, energy tunability and well defined source spectra make such a compact facility well suited for the XAS technique, especially in the hard X-ray region >15 keV. More details about the instrumentation, performance and capability of the MuCLS facility are given by Eggl *et al.* (2016 ▸) and Günther *et al.* (2020 ▸).

**Figure 2:**
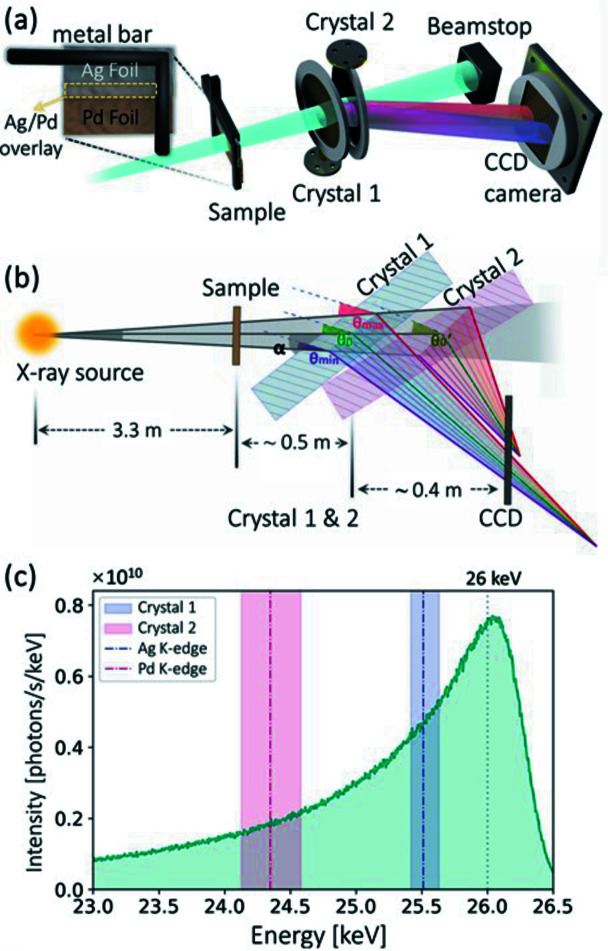
(*a*) Schematic illustration of the experimental setup and the sample. (*b*) Two slightly bent Laue crystals (denoted as Crystal 1 and Crystal 2) combined with the X-ray source divergence diffract two X-ray beams with different energy ranges. (*c*) The MuCLS source spectrum of 26 keV energy configuration (calibrated with the total flux at the time of the experiment). Red and blue regions indicate the maximum energy ranges of the X-ray beams diffracted by the Si(311) planes of Crystal 1 and Crystal 2. The difference in the energy range is due to the different bending radii of the crystals.

Figs. 2 ▸(*a*) and 2(*b*) show the two-color XAS experimental setup. The X-ray source of the MuCLS was tuned to a 26 keV energy configuration. The resulting spectrum is shown in Fig. 2 ▸(*c*), which has a full width at half-maximum (FWHM) of ∼1100 eV, *i.e.* a bandwidth of ∼4%. At the time of the experiment, the total X-ray flux exiting the source was ∼1.2 ×10^10^ photons s^−1^. The test sample was placed ∼3.3 m away from the X-ray source point. The sample consists of a 50 µm-thick Ag foil and a 25 µm-thick Pd foil, which are partially overlapping [see Figs. 2 ▸(*a*) and 3 ▸(*b*)]. Two silicon (Si) crystals (〈100〉 surface normal, primary flat horizontally aligned in the holder, 200 µm-thick, diameters of 100 mm, Sil’tronix Silicon Technologies, Archamps, France) are placed behind the sample, at a distance of ∼3.8 m from the X-ray source point. Both crystals are slightly bent in the meridional planes, the planes of energy dispersion. These crystals are rotated and aligned such that the Si(311) planes were under the Bragg condition. The first crystal (Crystal 1) was rotated by 34.2° relative to the incident beam direction [angle α in Fig. 2 ▸(*b*)], *i.e.* a Bragg angle of 9.0°. The second crystal was rotated by 33.9°, *i.e.* a Bragg angle of 8.7°. Combined with the ∼4 mrad X-ray divergence, two Laue beams are diffracted with different energy gradients in the horizontal direction. The diffracted Laue beam from Crystal 1 can cover an energy range suitable for Ag (*K*-edge 25.5 keV) XANES measurements. Crystal 2 is used for Pd (*K*-edge 24.4 keV) XANES in a similar way. The respective energy ranges are marked by the red and blue regions in Fig. 2 ▸(*c*). The two crystals are put in close proximity so that a single CCD detector can capture both diffracted Laue beams simultaneously. Consequently, no additional camera is needed, which also eliminates the need for camera syncrotronization. The detector is a XIMEA xiRAY CCD camera (model MH110XC-KK-FA, XIMEA GmbH, Münster, Germany) with a fiber-optically coupled Gd_2_O_2_S:Tb scintillator, which has a pixel size of 9 µm and a field of view (FOV) of 24 mm × 36 mm (horizontal × vertical).

The experiment was conducted in ambient conditions, as air does not significantly absorb high-energy X-rays. The only optical elements in the experiment were the two standard commercial silicon wafers, and thus the system is relatively efficient because additional optics may cause unwanted photon flux reduction. Furthermore, since no sophisticated crystal bender design or cumbersome X-ray focusing is needed, the installment and alignment of the setup are relatively feasible.

**Figure 3:**
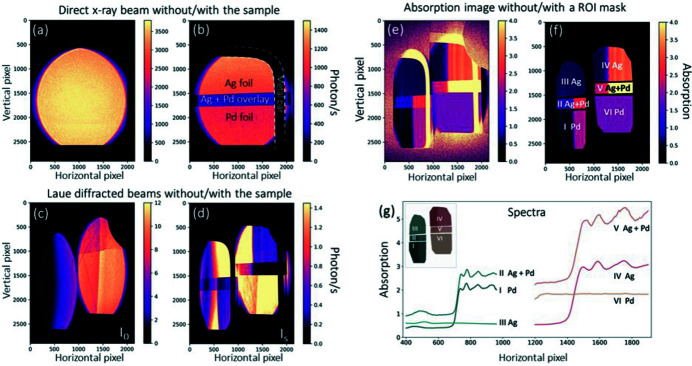
X-ray images recorded by the CCD camera (pixel size of 9 µm). (*a*) Direct X-ray beam without any Laue crystals or samples. (*b*) Direct X-ray beam without Laue crystals but with the sample. The components are labeled; see also the sample schematic shown in Fig. 2(*a*). An L-shaped metal bar is fixed to the sample for alignment. (*c*) Si(311) Laue diffracted X-ray beams with two crystals without the sample (denoted as I_0_). The broader beam with the higher X-ray intensity on the right is diffracted by Crystal 1. The artifacts visible in the X-ray beam are due to crystal defects and the X-ray transmission through the Crystal 2 holder. The left-hand beam is diffracted by Crystal 2. (*d*) The Si(311) Laue diffracted X-ray beams with both crystals and the sample (denoted as I_S_). The horizontal energy gradients can be visually distinguished, as the intensity fringes relate to the X-ray absorption fine structures. Also, the spatial information of the sample is well conserved, though some horizontally compressed distortions are present. (*e*) The absorption image is calculated pixel-to-pixel from I_0_ and I_S_ by Lambert–Beer’s law, which also removes the artifacts discussed for panel (*c*) from the data. (*f*) The absorption image is split into six regions of interest (ROIs). (*g*) By averaging all values in the ROIs along the vertical direction, the spectra can be generated as a function of pixels.

Fig. 3 ▸(*a*) shows a direct X-ray beam image without any X-ray optics or samples, captured by the CCD camera at a distance (along the incident X-ray direction) of ∼4.2 m from the source. The exposure time for this image was 0.1 s. The intensity is homogeneous throughout the beam and remained stable during the experiment. The 4.0 mrad × 4.5 mrad (horizontal × vertical) divergence gives rise to an elliptical beam with a size of 1.7 cm × 1.9 cm (horizontal × vertical) on the camera. The lower part of the beam is intercepted by a specifically designed X-ray beam monitor (Günther *et al.*, 2019 ▸), which records and stabilizes X-ray beam properties such as X-ray flux and source size during the experiment. Fig. 3 ▸(*b*) shows a direct X-ray projection image of the sample without any X-ray optics. An L-shaped metal bar with a diameter of 2 mm is attached to the sample for alignment, indicated by the white dashed line. Due to similar *K*-edge absorption edge energies and the polychromacity of the direct X-rays, it is impossible to unambiguously distinguish the two different metals solely from this projection image.

Figs. 3 ▸(*c*) and 3 ▸(*d*) show the Laue diffracted beams, without (denoted as I_0_) and with the sample (denoted as I_S_). The diffracted beams have much lower intensities than the direct beam. The first crystal and the second crystal diffract ∼0.12% and 0.03% of the total flux (1.2 × 10^10^ photons s^−1^). The exposure time was 30 s for each frame, and 20 frames were averaged for each of these two images for better image quality as the proof-of-principle demonstration. Figures S1 and S2 of the supporting information further show that the acquisition time can be shortened to 1 min (30 s for I_S_ and 30 s for I_0_). The acquisition can be even faster because the exposure for the intense flatfield (I_0_) can be shortened and the flux at the time of the experiment was not in the most optimal condition (in which flux can surpass 2 × 10^10^ photons s^−1^). A median filter (Huang *et al.*, 1979 ▸) with a kernel size of 3 × 3 pixels is applied to the images to remove impulse noise. The Laue beams have a similar vertical length but narrower horizontal width than the direct X-ray beam, caused by a combined effect of the asymmetry angle of the lattice plane and crystal bending in the Laue geometry. The left-hand beam is diffracted by Crystal 2, which covers a maximum horizontal size of ∼750 pixels and an energy range of ∼460 eV in the Pd *K*-edge XANES regime. In contrast, the one on the right is diffracted by Crystal 1 and matches the Ag *K*-edge XANES regime. It covers a maximum horizontal size of ∼960 pixels and an energy range of ∼220 eV. The variations in beam sizes and energy ranges are due to the different curvatures of the crystals. Both crystals are slightly bent in a diverging manner such that the convex sides of the crystals face the detector, causing a slight increase of the beam size and a reduction of the total energy spread compared with a perfectly flat crystal. As a result, the energy resolution can be slightly improved in the current geometry. For this reason, the energy resolution of the spectra generated from Crystal 1, which has a smaller bending radius, is better than that from Crystal 2.

To retrieve the final XAS spectra, an absorption image [Fig. 3 ▸(*e*)] is calculated according to Lambert–Beer’s law. Several polygonal regions of interest (ROIs) are selected [Fig. 3 ▸(*f*)], covering the respective parts of the sample for the two different energy ranges. For example, ROIs III and IV both cover the same part of the Ag foil, but, unlike ROI IV, no absorption structure is visible in ROI III, as the energy range for ROI III is below the Ag *K*-edge.

Fig. 3 ▸(*g*) shows X-ray absorption spectra as a function of pixel coordinates, calculated by averaging all the values in these ROIs vertically. When the absorption features are tilted or distorted [see example in the supporting information of Huang *et al.* (2020 ▸)], additional post-processing such as image rotation or image warping is needed to straighten the features. For energy calibration, a spectrum is recorded for a reference system of the element of interest, *i.e.* pure Ag or Pd foils in this case. Pixel positions are then calibrated to X-ray energies by matching the MuCLS spectrum to energy-calibrated synchrotron standard data for the same element. This is done by extracting spectral features in both spectra – local extrema and edge jump points – and then performing a second-order fit. The identical energy calibrations can be used for other samples containing Ag or Pd measured under the same conditions.

## 3. Results and discussion

**Figure 4:**
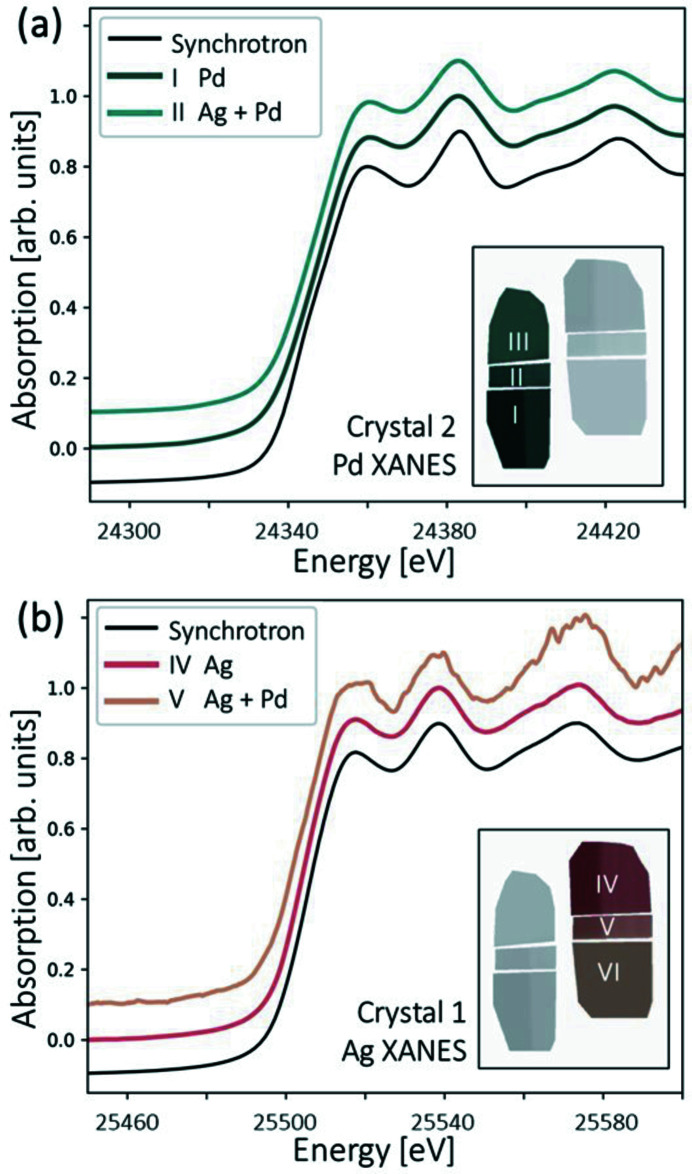
(*a*) Normalized Pd XANES spectra generated from ROIs I and II in Fig. 3 ▸(*f*). (*b*) Normalized Ag XANES spectra generated from ROIs IV and V. Since spectra generated from ROIs III and VI have no visible spectral features, they are not plotted here.

Fig. 4 ▸ shows the final XANES spectra of ROIs I and II as well as those of ROIs IV and V. Both ROIs II and V spectra correspond to the same part of the sample (the Ag and Pd overlay). The total attenuation of the Ag and Pd overlay is relatively high above 25.5 keV, resulting in low signal transmission. For this reason, the Ag spectrum obtained from ROI V is noisier. This can be improved by adjusting the thickness of the sample.

The final energy resolution of the XAS setup can be evaluated by a convolution of several factors, including the X-ray source size, the spatial resolution of the detector, the intrinsic crystal bandwidth, the Borrmann fan of the Laue crystal, and an additional Gaussian filter used in the post-processing. The calculated energy resolutions are 6.8 eV and 8.0 eV for Ag and Pd measurements, respectively. Both values are close to the *K*-hole lifetime broadening of Ag and Pd (Campbell & Papp, 2001 ▸). Except for the noisier ROI IV spectrum, the obtained spectra are in good agreement with synchrotron data.

The bending radii for the two crystals are estimated to be ∼10 m and ∼27 m for Crystal 1 and Crystal 2, respectively, determined by the difference between the experimental diffracted energy range and the theoretical diffracted energy range. The bending radius of each crystal is tunable by adjusting the forces applied to its edges in the 3D-printed crystal holder. Apart from tuning crystal bending, other factors can also be used to tune beam properties, such as asymmetry angles and diffraction orders.

Unlike systems involving only one polychromator, in this setup the energy range and energy resolution of the X-ray beams diffracted by the two crystals can be independently tuned to suit the requirements of the experiment. The diffracted X-ray beams’ positions are adjustable, allowing two beams to be imaged on the same CCD camera. The energy ranges of the diffracted beams can also be extended to measure extended X-ray absorption fine structure (EXAFS) by switching diffraction planes to Si(111) (Huang *et al.*, 2020 ▸). However, as the MuCLS source spectrum has a bandwidth of just 3–5% and is tunable from 15 keV to 35 keV, the absorption edges of multiple elements need to be in a relatively narrow energy window. The setup is applicable to a specific range of bimetallic systems. Besides the Ag–Pd systems, it may find applications in bimetallic systems involving Nb–Mo (Schwartz *et al.*, 2000 ▸), Rh–Pd (Soler *et al.*, 2019 ▸) and Sb–Te (Nagulapati *et al.*, 2020 ▸) pairs, among others. Another limitation of the current setup is that the X-ray footprint on the sample is relatively large. As a result, samples need to be prepared in the form of homogeneous pellets.

The setup geometry we propose here can be further adapted to other ICS facilities (Cardarelli *et al.*, 2020 ▸; Chi *et al.*, 2017 ▸; Deitrick *et al.*, 2018 ▸; Dupraz *et al.*, 2020 ▸; Graves *et al.*, 2014 ▸; Vaccarezza *et al.*, 2016 ▸). In particular, X-ray sources that aim to provide a two-color spectrum (Cardarelli *et al.*, 2020 ▸; Drebot *et al.*, 2017 ▸) may overcome the limitation that absorption edges of multiple elements need to be in close proximity. As the ICS source used here produces divergent X-rays with a polychromatic spectrum, the two-color setup may be extended to bending magnet beamlines or more commercially available laboratory sources, such as X-ray tubes with high-flux bremsstrahlung. For example, a micro-focus metaljet source (Metaljet E1+ 160 kV, Excillum AB, Sweden) provides a monochromatic flux of 6–7 × 10^8^ photons s^−1^ eV^−1^ sterad^−1^ for its bremsstrahlung at an energy of about 25 keV (according to the spectrum provided by its website https://www.excillum.com/products/metaljet/metaljet-e1-160-kv/). Consequently, by a direct and rough estimate, the acquisition time may be prolonged by a factor of two to three orders of magnitude for the metaljet sources or even longer for less powerful X-ray tubes. Nevertheless, X-ray tube-based setups may have good potential in studying mechanisms and dynamics involved in slow reactions.

## 4. Conclusion

In summary, we propose a simultaneous two-color XAS setup for a polychromatic, divergent X-ray source, here demonstrated at a laboratory ICS source facility. We carried out a proof-of-principle experiment on an Ag–Pd sample and obtained Ag and Pd XANES spectra with promising spectral quality. The setup, mainly using two Laue crystals, is straightforward to implement and enables independent tuning of the energy range and energy resolution for different elements. Furthermore, the diffracted Laue beams, which contain both energy-resolved and spatially resolved information, open up possibilities of simultaneous spectral or spectro-imaging applications.

## Supplementary Material

Figures S 1 and S2 of the supporting information. DOI: 10.1107/S1600577521009437/ok5051sup1.pdf
