# Supplementary material for: Simultaneous two-color X-ray absorption spectroscopy using Laue crystals at an inverse-compton scattering X-ray facility
Source: J Synchrotron Radiat. 2021 Nov 3;28(Pt 6):1874–80. doi: 10.1107/S1600577521009437 (PMC8570203; doi:10.1107/S1600577521009437)
Supplement: Supplementary file 1 [file s-28-01874-sup1.pdf]

JOURNAL OF SYNCHROTRON RADIATION

Volume 29 (2022)

Supporting information for article:

Simultaneous two-color X-ray absorption spectroscopy using Laue crystals at an inverse-compton scattering X-ray facility

Juanjuan Huang, Benedikt Günther, Klaus Achterhold, Martin Dierolf and Franz Pfeiffer

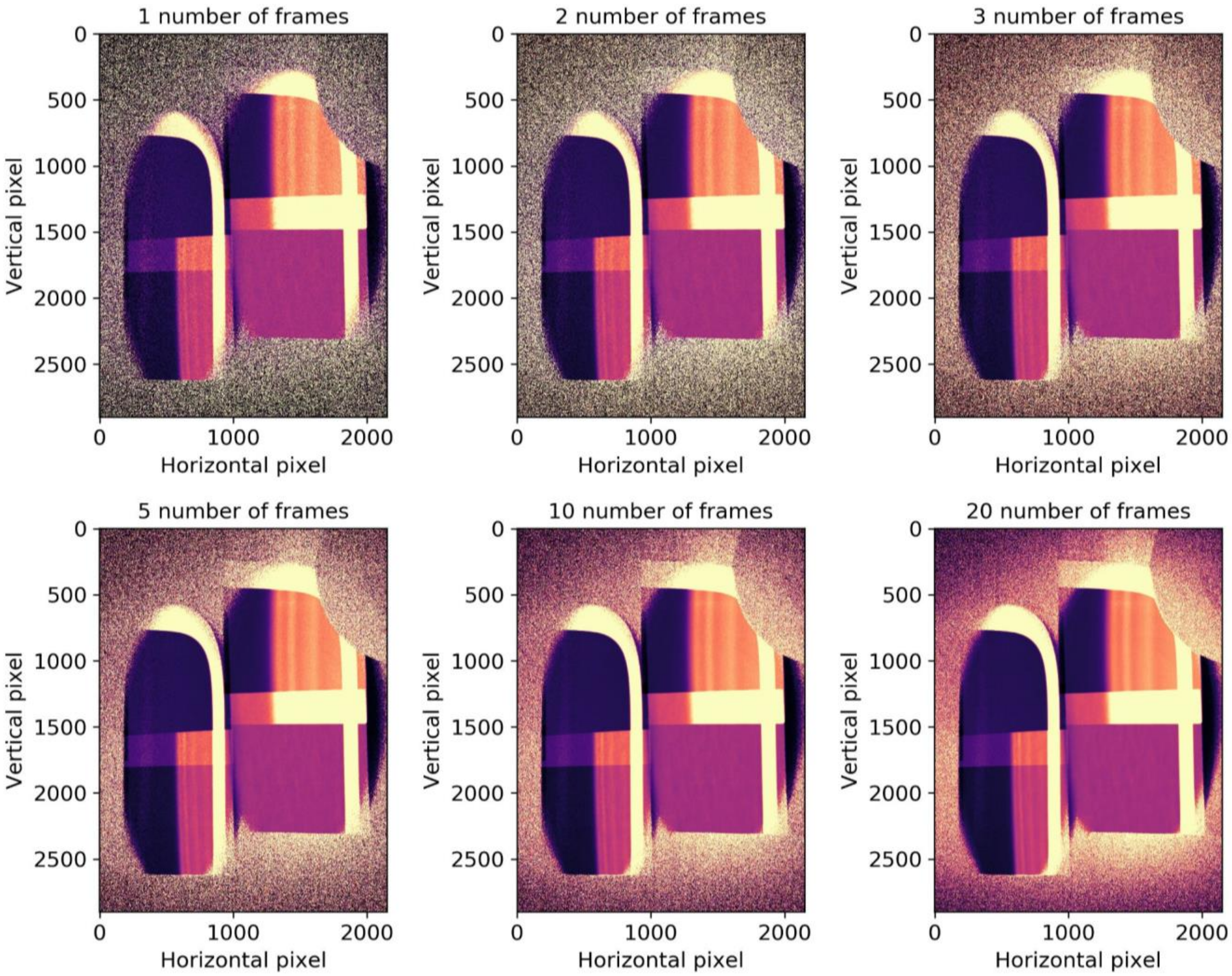

Figure S1 Absorption images of averaging different number of frames (the exposure time was 30 s/frame for both the transmitted image Is and I_0)

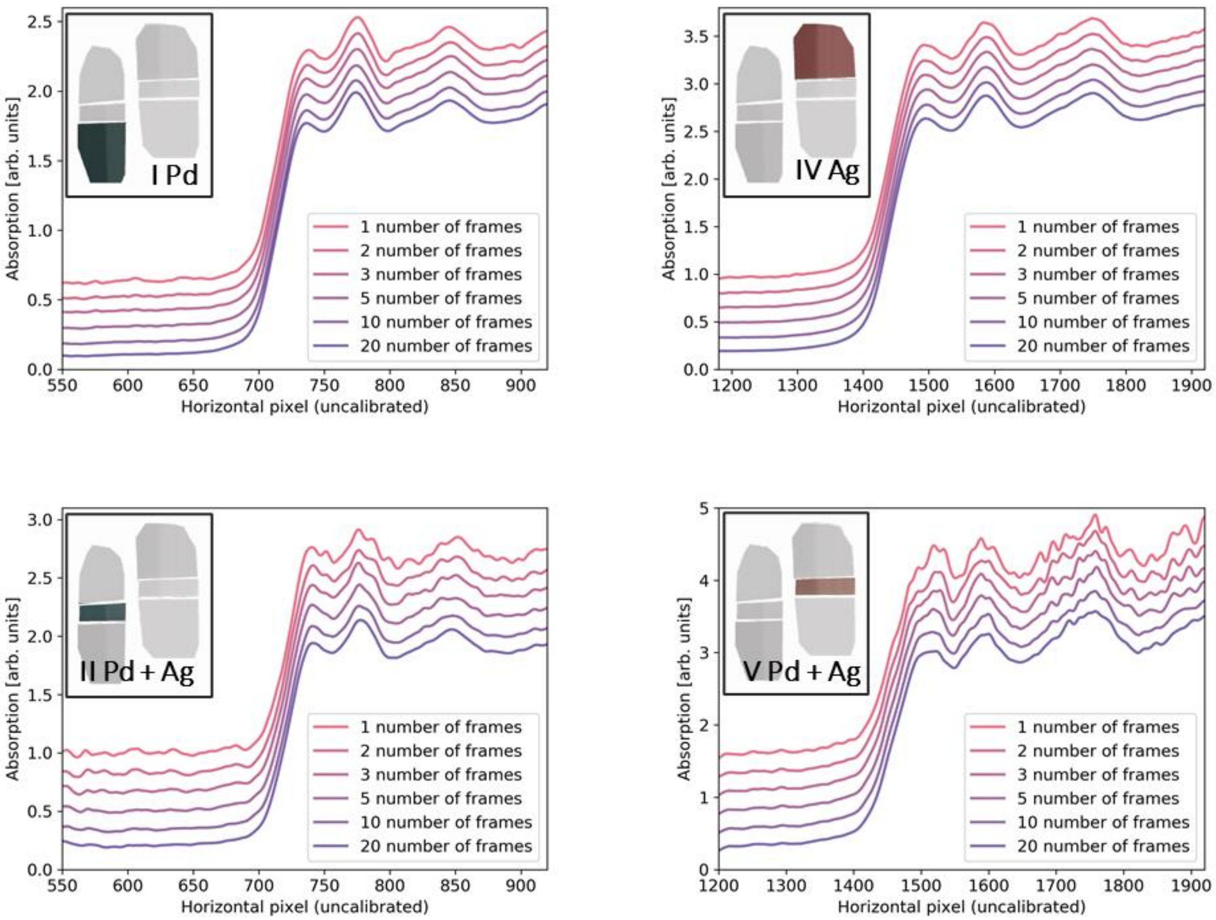

Figure S2 Corresponding spectra generated from averaging different numbers of frames (uncalibrated)
